# Supplementary material for: Transcriptome and Gene Co-Expression Network Analysis Identifying Differentially Expressed Genes and Signal Pathways Involved in the Height Development of Banana (Musa spp.)
Source: Int J Mol Sci. 2023 Jan 30;24(3):2628. doi: 10.3390/ijms24032628 (PMC9917265; doi:10.3390/ijms24032628)
Supplement: Supplementary file 1 [file ijms-24-02628-s001.zip › ijms-2117215-supplementary.pdf]

Supplementary Data

Transcriptome and Gene co-expression network analysis identifying differentially expressed genes and signal pathways involved in the height development of banana (*Musa* spp.)

Bingyu Cai [1,2], Yufeng Chen [2], Miaomiao Cao [1,2], Junting Feng [2], Yuqi Li [2,3], Liu Yan [2,3], Yongzan Wei [2], Yankun Zhao [2], Yixian Xie [2], Wei Wang [2], Jianghui Xie [2]

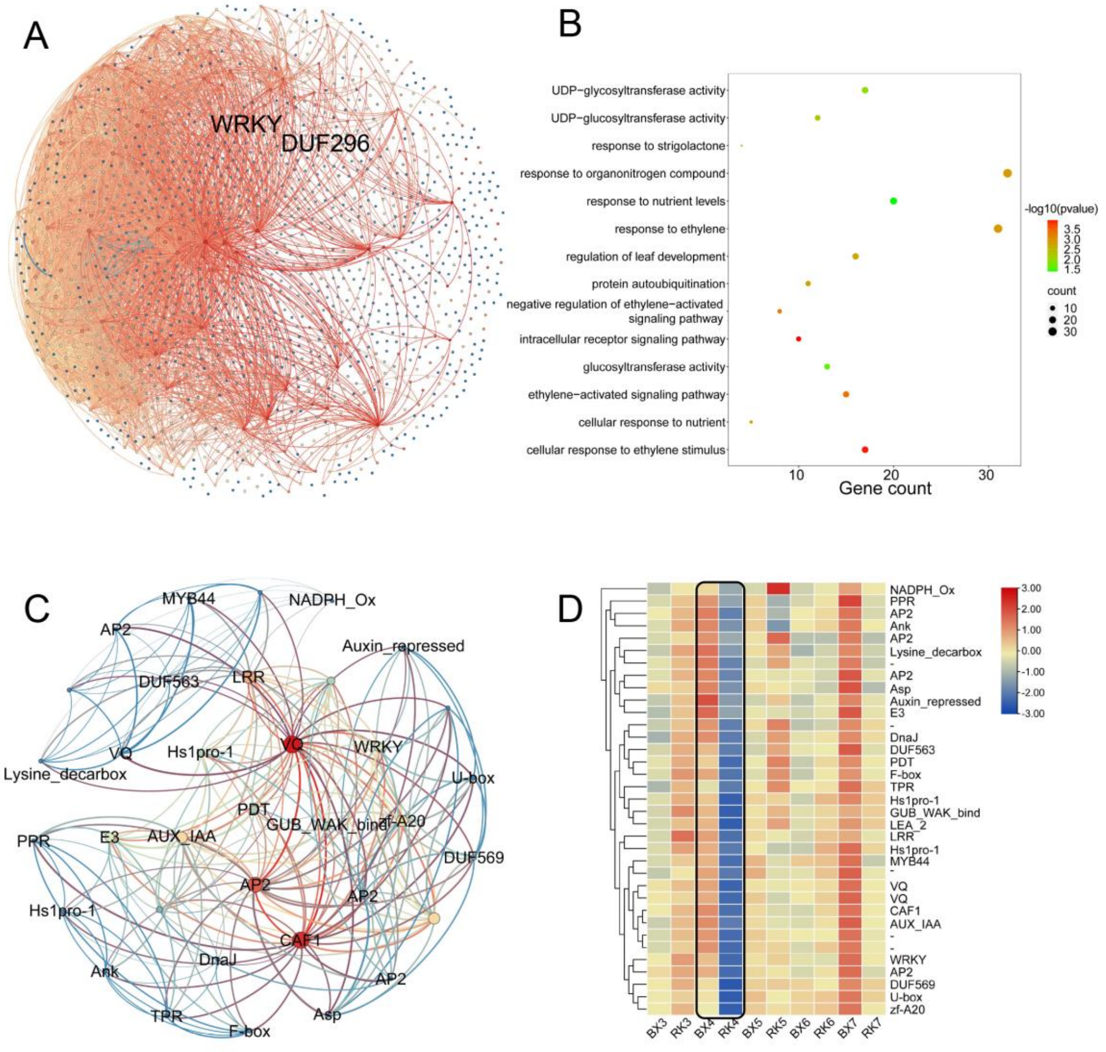

Figure S1. Co-expression network analysis of genes in the salmon module. (A) Co-expression network of genes in the salmon module. The size of nodes represented the numbers of genes connected with others. The width of edges represented the weight between two genes connected; (B) GO enrichment analysis of the white module. The size of the point represented the gene numbers; (C) Merging network of hub genes using 12 methods; (D) Expression of hub genes from the third week to the seventh week. The color scale ranging from green and red indicated the expression levels of gene.

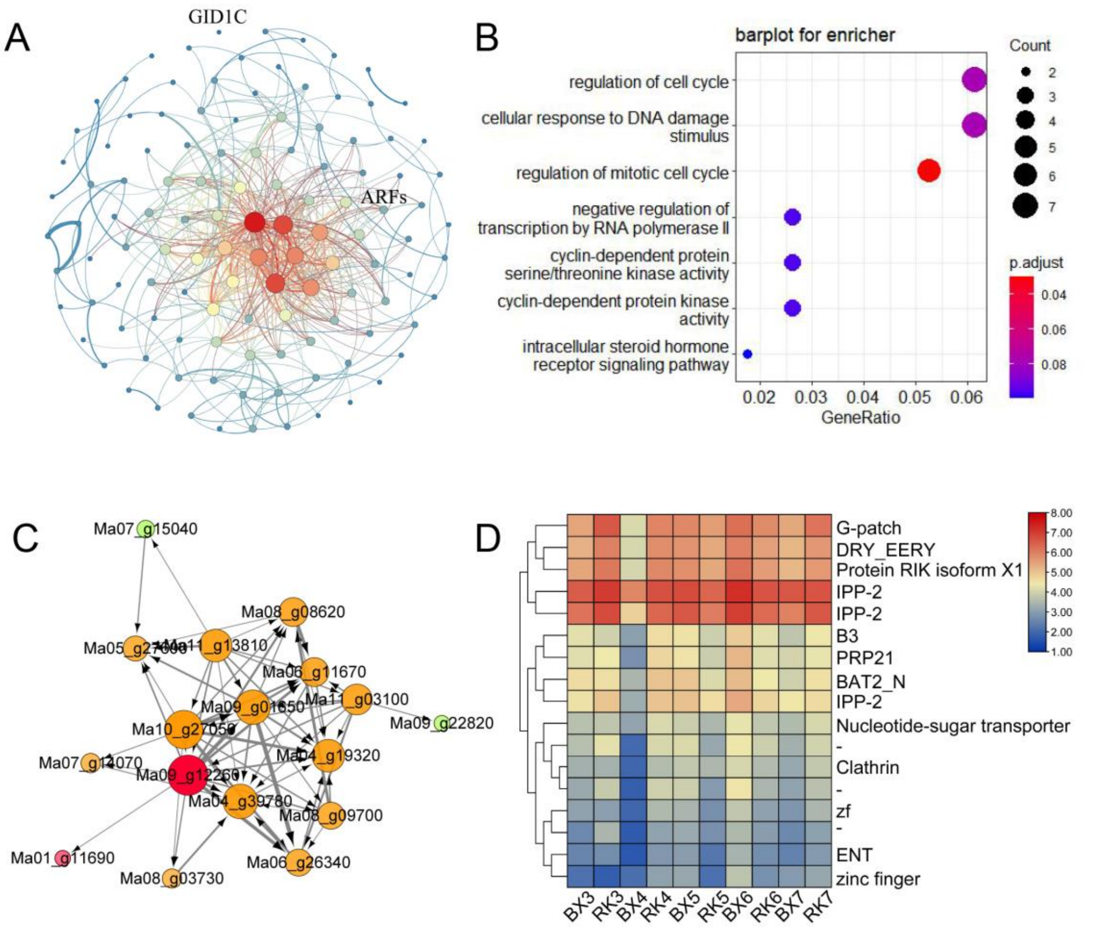

Figure S2. Co-expression network analysis of genes in the white module. (A) Co-expression network of genes in the white module. The size of nodes represented the numbers of genes connected with others. The width of edges represented the weight between two genes connected; (B) GO enrichment analysis of the white module. The size of the point represented the gene numbers; (C) Merging network of hub genes using 12 methods; (D) Expression of hub genes from the third week to the seventh week. The color scale ranging from green and red indicated the expression levels of gene.

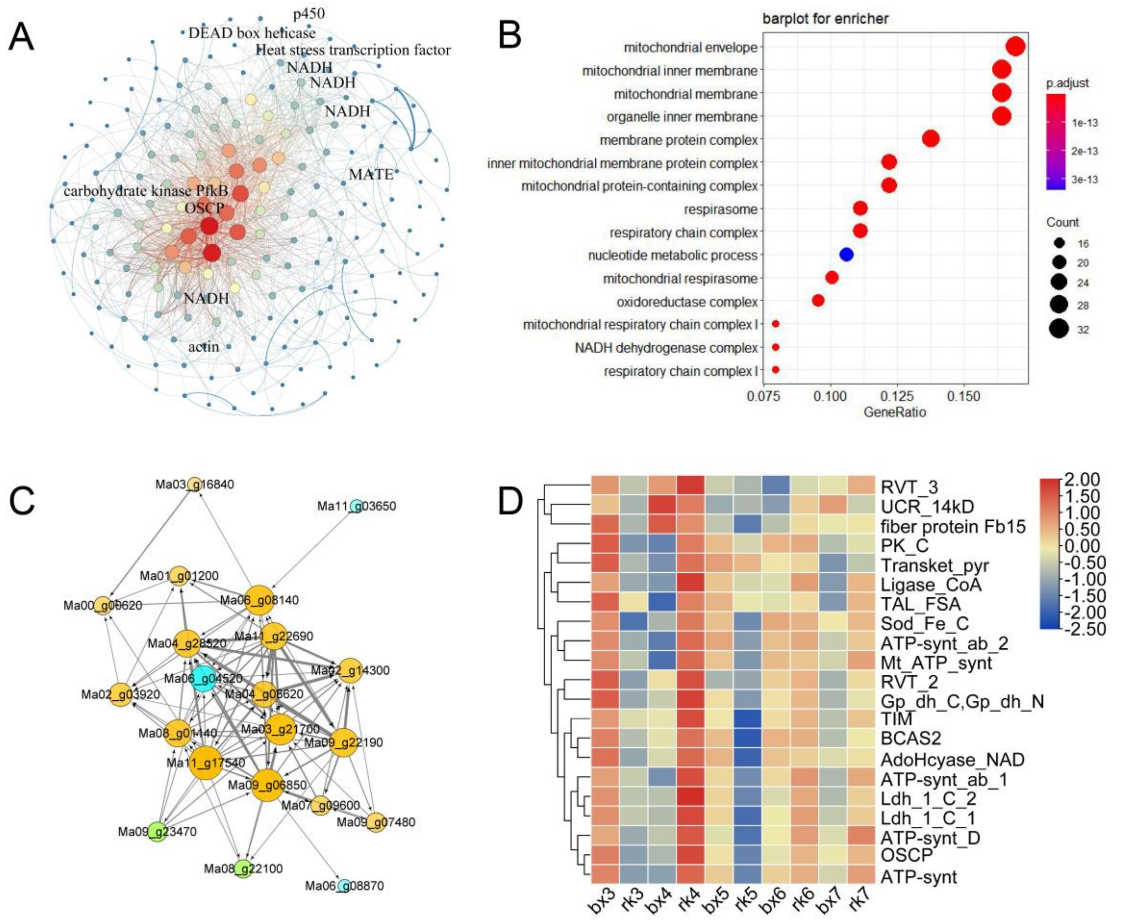

Figure S3. Co-expression network analysis of midnightblue module. (A) Network of white module. The size of nodes represents the number of connections with other. The width of edges represents the weight between two genes connected; (B) GO enrichment analysis of white module. The size of the point represents the number of genes; (C) Merging network of hub genes by 12 Methods; (D) Expression of hub genes from the third to the seventh weeks. The colour scale range from green and red indicates the level of gene expression.

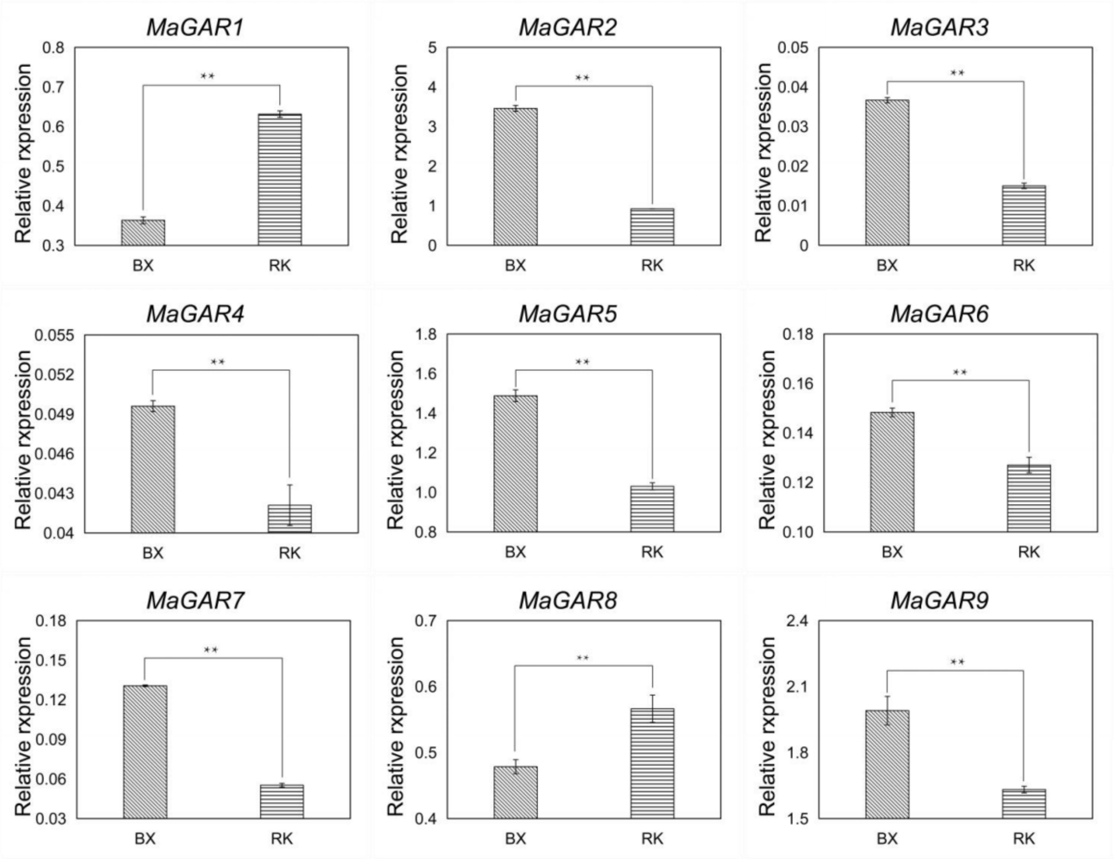

Figure S4. Expression levels of candidate genes in the fourth week using qRT-PCR. Data were means of three biological repetitions. Each bar represents mean ± SE and asterisks (**) indicate significant differences (Student's t-test, $P < 0.01$).

Table S1. The filtered results of sequence data.

Sample	raw Data Reads	clean Data Reads	Q20	Q30	GC content
BX3-1	40,123,622	40,123,574	98.37	95.47	50.32
BX3-2	40,470,234	40,426,014	97.76	94.04	50.61
BX3-3	40,270,654	40,225,742	98.25	95.14	50.49
BX4-1	40,330,502	40,304,644	98.06	95.1	53.42
BX4-2	40,388,898	40,362,402	98	94.87	53.76
BX4-3	42,475,682	42,437,750	98	94.97	53.45
BX5-1	40,250,438	40,250,340	98.3	95.21	49.27
BX5-2	40,091,996	40,091,978	98.21	94.8	49.6
BX5-3	38,585,760	38,585,726	98.27	95.14	49.54
BX6-1	38,773,434	38,773,422	98.39	95.37	48.74
BX6-2	41,308,904	41,308,844	97.73	93.85	48.75
BX6-3	40,331,090	40,331,038	98.37	95.34	49.03
BX7-1	40,427,308	40,427,230	98.2	95.16	51.36
BX7-2	40,176,836	40,147,430	98.13	95.03	51.8
BX7-3	41,389,188	41,389,114	98.22	95.08	51.38
RK3-1	40,036,206	40,036,168	98.14	94.57	50.47
RK3-2	52,147,388	52,147,316	98.05	94.81	48.54
RK3-3	42,836,002	42,835,942	98.48	95.73	50.07
RK4-1	40,525,352	40,525,300	98.55	95.71	48.26
RK4-2	40,065,154	40,065,132	98.46	95.53	49.46
RK4-3	41,441,704	41,441,676	98.47	95.47	47.91
RK5-1	40,475,146	40,475,084	98.43	95.64	50.38
RK5-2	39,993,384	39,993,328	98.29	95.29	49.64
RK5-3	44,132,554	44,132,510	98.43	95.57	50.66
RK6-1	40,512,578	40,512,538	98.43	95.48	50.01
RK6-2	40,183,416	40,183,358	98.41	95.51	50.73
RK6-3	40,400,078	40,400,024	98.44	95.52	50.13
RK7-1	39,980,726	39,980,682	98.4	95.44	49.02
RK7-2	38,631,042	38,630,996	98.54	95.73	49.14
RK7-3	39,292,872	39,292,816	98.41	95.47	49.74

Table S2. The alignment result with the banana reference genome.

sample	number of mapped reads	mapped rate	number of duplicated reads (estimated)	duplication rate	mean coverage Data	std coverage Data
BX3-1	41,510,058	93.47%	31,355,187	60.93%	55.2224X	557.6396X
BX3-2	41,469,217	92.74%	31,658,547	61.63%	54.4823X	559.2563X
BX3-3	41,729,175	93.54%	31,601,955	60.65%	54.7896X	560.6259X
BX4-1	43,633,078	93.52%	37,571,678	67.97%	90.0251X	1,012.7183X
BX4-2	43,429,278	93.52%	37,174,197	66.57%	75.3281X	980.8672X
BX4-3	45,182,303	93.19%	38,992,945	70.05%	79.5474X	935.1825X
BX5-1	39,099,188	92.61%	28,063,492	60.98%	43.9785X	219.0462X
BX5-2	39,243,266	93%	28,490,384	61.68%	44.4772X	262.2725X
BX5-3	37,543,217	92.90%	27,055,405	61.38%	42.939X	217.8257X
BX6-1	38,308,961	92.90%	27,371,549	61.01%	45.1055X	300.9743X
BX6-2	39,899,319	92.18%	28,586,395	62.01%	46.8995X	216.4949X
BX6-3	39,361,257	92.86%	28,042,544	60.54%	47.2981X	230.945X
BX7-1	42,235,682	93.22%	33,801,955	63.23%	57.04X	667.9701X
BX7-2	41,894,699	93.26%	33,718,067	63.96%	54.4783X	680.9591X
BX7-3	43,399,116	93.26%	34,384,050	61.78%	66.4598X	706.7178X
RK3-1	41,045,261	92.97%	32,167,434	62.96%	56.3691X	574.7031X
RK3-2	52,931,320	90.17%	44,339,977	71.46%	74.2937X	960.3426X
RK3-3	44,085,301	93.12%	34,807,087	65.01%	54.0589X	604.6337X
RK4-1	39,048,883	93.02%	27,786,143	61.91%	49.9527X	150.5702X
RK4-2	39,852,269	93.29%	29,537,939	62.18%	50.5114X	334.9944X
RK4-3	39,715,768	92.55%	28,682,769	64.45%	50.5811X	148.3321X
RK5-1	42,099,289	93.02%	33,843,994	65.56%	54.534X	613.6916X

RK5-2	41,117,744	92.20%	32,833,413	63.70%	53.982X	621.3479X
RK5-3	44,900,105	93.45%	35,577,292	66.19%	52.6817X	530.8676X
RK6-1	39,449,868	92.98%	29,405,413	62.68%	46.0123X	233.927X
RK6-2	38,909,144	93.23%	29,598,957	65.33%	45.5394X	197.7415X
RK6-3	39,378,389	93.28%	28,963,539	62.23%	46.2079X	199.8666X
RK7-1	38,165,283	92.73%	28,302,782	65.47%	46.9445X	145.3664X
RK7-2	37,148,132	93.14%	26,535,850	62.51%	45.1312X	144.0431X
RK7-3	37,818,277	93.27%	28,193,287	65.30%	45.5706X	168.9739X

Table S3. The primers used by qRT-PCR.

**Primer id**	**Forward primer**	**Reverse primer**	**length**	**Tm**
MaGAR1	GAAGCCAAACATGCGTCGAA	GGCCACAAGAGAGAGCCATC	188	60
MaGAR2	CTGAGCTCTTAGACTCGCCG	TGCGATGGTTTGGAACGAGA	173	60
MaGAR3	GGAGGACTCGAGTGCAGAAG	AGTCCCGGTAATGAGCTTGC	183	60
MaGAR4	TCTGGTTCAGGACTGGCAAC	GAGCCGACGAAGTCGTGTAT	162	60
MaGAR5	CCAGTAAGCTGCTTTCACGC	TAGTTGACGGACACGACGAC	223	60
MaGAR6	CCCCTCGAGGAGCTTCCTTT	CCGGTAGACGAGCTTCACAT	129	60
MaGAR7	ATGCTCCAGGAACAAGCCAC	AGGACGGACCTGCTAACTCT	151	60
MaGAR8	TCTCGCCTGTTCATCGACCT	TCGGCGTAGGAGTAGTAGGTT	158	60
MaGAR9	CGTCGTGTCCGTCAATTACC	CAGTTTCCGCCAAACATGGG	252	60
Actin	TGGTATGGAAGCCGCTGGTA	TCTGCTGGAATGTGCTGAGG	263	60
